# Cause and Effect; Prevention or Cure

**Published:** 1913-10

**Authors:** 


### EDITORIAL DEPARTMENT.

DR. F. E. DANIEL, Editor DR. R. H. L. BIBB, Associate Editor

DEPARTMENTS { EUGENICS: Drs. M. Duggan and T. Y. Hull.

WOMAN'S DEPARTMENT: Mrs. F. E. Daniel.

Cause and Effect; Prevention or Cure.—State medicine is the application in the aggregate, of the principles of medical and sanitary science to the prevention, cure, mitigation or relief of evils which affect the social body, and the prevention of those evils to posterity. It bears the same relation to the State or society that the individual physician bears to his clientelle; and embraces, of course, measures of prophylaxis against future ills as against existing evils. For illustration, any measure calculated to improve the race—we will say—restrictions upon marriage, limiting the privilege to the fit, or sterilization of natural criminals, or insane criminals, or the criminal insane, to cut off succession, are as much within the scope of its beneficent functions as is quarantine against disease.

The State owes no higher duty to posterity than to protect it against a multiplication of those evils we now deplore, and are ineffectually battling against.

The Medical profession has ever been characterized by humanity and benevolence. The profession do all in their power to relieve suffering. Our grand hospitals and asylums are monuments to the benevolence and unselfishness of medicine. Yet it seems to be, after all, a false philanthropy, as it enables the afflicted ones to live on and get more children for the next generation to care for. Thus evil comes out of good, and our best intentions react to the ultimate detriment of society. By practical charity, alms giving, and the tender care for the defectives and diseased, the operation of nature's laws is defeated, and the unfit survive—and breed and multiply like flies.

On the subject of marriage, Judge C. H. Reeves, of Plymouth, Indiana, in a work called "The Prison Question," the most logical exposition of the subject I have ever seen, says:

"In regulating marriage, the law says that none shall marry within the third degree of consanguinity, and in some States the fourth, because marriage between near blood relations is likely to produce offspring deformed or diseased, physically and mentally. Insane and idiots shall not marry, because they cannot make a contract, and because of heredity tendency to produce idiots and insanity. It makes it a crime to marry in any of these cases. In this it aims to prevent degenerate offspring and protect individuals and society against the evils that would attend such offspring. * * *

"But if the vilest mortal that can live—one not in these classes—sees proper to marry, the law issues the license for the asking, taking a fee, makes a record, and leaves the offspring and society to shift for themselves in the best way they can. The confirmed inebriate, the weak-minded and semi-idiotic, the confirmed criminal, the offspring of the half-witted and insane, if lucid at the time—the incurably diseased, the scrofulitic, the syphilitic, the hereditary pauper, the depraved and reckless—even paupers while in the poorhouse, and criminals while in jail, are in every way encouraged, given license, and are protected by the law. No thought is taken for the unfortunate offspring, nor for the body politic or social, and the irreparable evils that must fall upon all. The church adds its sanction, and its ministers aid in making these civil contracts, by performing the ceremony with benediction and prayer.

"If it is wise to prohibit polygamy, marriage between near relations, between the insane and idiotic, because of heredity and transmissions of evils, it is equally wise to prohibit it in all cases where like evils may follow. If the law has the power to prohibit and punish violations in one case, it has equal right in all others.

"There is an endless procession of children from all these sources coming into the mass of population to live lives of crime, immorality, want, suffering, misfortune and degradation, transmitting the taint in constantly ever widening streams, generation after generation, with the ultimate certainty of the deterioration of the race, and final irreparable degeneracy.

"It seems to me that there is a moral obliquity that affects the entire mass of political, social and religious leaders and teachers on the subject here being considered. When we analyze the views and actions throughout, the glaring inconsistency and unreasonableness that seems to fill them has no parallel in any other matter seriously affecting individual and the public welfare. Among the first is a false modesty, that is shocked by any allusions to the most evident and debasing facts that stare everybody in the face on all sides; that rub everybody at every turn.

"The church devotes its time and energies to prove that every human body possesses an immortal spiritual body, that is liable to future torture unless it be made perfect in morals and truth, and that must be done while it remains in its mortal shell. It pleads and raves for prohibition of liquors and tobacco, for forced observance of Sunday, for forced attendance on schools, for recognition of God, Christ and the Protestant religion in the civil constitutions, and for sundry other restraints and commands, with penalties, in order to save these imperiled souls. Reformers go about the land devising ways and means to educate, civilize, provide for and elevate the ignorant, the degraded, the poverty stricken that pervade every plane of human action, and wander in and out among the people everywhere. And yet these, with general society added, hold up their hands before their faces in horror, if some honest soul who has truth for a guide, calls to them to look, and points them to the source of the evils they are battling with, and tells them they are responsible for it all, for the law is only their united will in statutory phraseology. That it is the result of their voluntary blindness and false conception of civil, moral and religious duties. That they are seeking to deal with evil conditions alone, instead of the causes of them, and while trying to mitigate the evils in the results, are supporting, increasing and enlarging the causes. That on every other plane of action they recognize and deal with the causes; but with men and women they ignore the causes and battle with results alone. That they regard domestic brutes as of more importance than they do human beings."

But, so long as newspapers are run to make money, and not in the aid of humanity or science, or for the dissemination of truth, or, as is often the case, subsidized by the whiskey ring, it were idle to preach against it; they will never aid science in any reform in the interest of truth, humanity or religion. No paper can be found with the honesty and independence to advocate any measure or reform—or to disseminate any truth in the interest of humanity that conflicts with that interest.

